# Soluble Epoxide Hydrolase Inhibitory Activity of Selaginellin Derivatives from *Selaginella* *tamariscina*

**DOI:** 10.3390/molecules201219774

**Published:** 2015-12-02

**Authors:** Jang Hoon Kim, Chong Woon Cho, Bui Huu Tai, Seo Young Yang, Gug-seoun Choi, Jong Seong Kang, Young Ho Kim

**Affiliations:** 1 College of Pharmacy, Chungnam National University, Daejeon 305-764, Korea; oasis5325@lycos.co.kr (J.H.K.); chongw113@cnu.ac.kr (C.W.C.); bhtaiich@gmail.com (B.H.T.); ysy1008@chol.com (S.Y.Y.); 2 Department of Horticultural Environment, National Institute of Horticultural and Herbal Science, RDA, Wanju-gun, Jeollabuk-do 595-890, Korea; choigs@korea.kr; 3 Institute of Marine Biochemistry, Vietnam Academy of Science and Technology, 18-Hoang Quoc Viet, Caugiay, Hanoi 364-545, Vietnam

**Keywords:** *Selaginella tamariscina*, Selaginellaceae, selaginellin, soluble epoxide hydrolase, non-competitive inhibition

## Abstract

Selaginellin derivatives **1**–**3** isolated from *Selaginella* *tamariscina* were evaluated for their inhibition of soluble epoxide hydrolase (sEH) to demonstrate their potential for the treatment of cardiovascular disease. All selaginellin derivatives (**1**–**3**) inhibited sEH enzymatic activity and PHOME hydrolysis, in a dose-dependent manner, with IC_50_ values of 3.1 ± 0.1, 8.2 ± 2.2, and 4.2 ± 0.2 μM, respectively. We further determined that the derivatives function as non-competitive inhibitors. Moreover, the predicted that binding sites and interaction between **1**–**3** and sEH were solved by docking simulations. According to quantitative analysis, **1**–**3** were confirmed to have high content in the roots of *S. tamariscina*; among them, selaginellin **3** exhibited the highest content of 189.3 ± 0.0 μg/g.

## 1. Introduction

*Selaginella tamariscina*, belonging to the genus Selaginellaceae, is used as a Chinese and Korean herbal medicine to promote blood circulation and for the treatment of cancer, hepatitis, diabetes, and skin diseases [1]. Fifteen derivatives, selaginellin A-N, have been isolated from *Selaginella* since 2007 [2]. Their rare structure includes a carbon scaffold containing pigments similar to the alkynylphenol and *para*-quinone methide skeleton [1]. Due to characteristic features of this structure, their color changes from red to pink within a pH range of 1.5 to 1, and from red to purple within a pH range of 7.5 to 8.0. In addition, their UV-Vis absorption intensities change according to the pH [2]. In biosynthesis pathways, selaginellin derivatives are constructed from the basic structure of orsellinic acid, and produced by type III polyketide synthases (type III PKS) using four malonyl-CoA [3]. Their inhibitory effects on the expression of HeLa cells, MCF-7, and free radical scavenging, as well as their protective effects against cytotoxicity and apoptosis, have been shown in differentiated PC12 cells [1,2,4].

Epoxyeicosatrienoic acid (EET) regioisomers 5,6-EET, 8,9-EET, 11,12-EET, and 14,15-EET, were produced from arachidonic acid using cytochrome *P*-450 (CYP) [5,6]. A previous study reported that human recombinant CYP generates an equal amount of 11,12-EET and 14,15-EET, but little 5,6-EET and 8,9-EET [6,7,8,9]. These regioisomers consistently exhibit vasodilator activity, thus opposing the actions of vasoconstrictors, which contributes to cardiovascular disease. An increase in the amount of endothelial EETs has also been attributed to cardiovascular disease [6,7,8,9].

Soluble epoxide hydrolase (sEH) is widely present in mammalian tissues. sEH consists of a homodimer of ~62-kDa monomeric units, with a C-terminal domain of ~35 kDa, which provides its epoxide hydrolase activity; this is responsible for the hydrolysis function that converts the epoxide to the corresponding diol [5,10]. For example, 14,15-EETs are metabolized as corresponding 14,15-dihydroxyeicosatrienoic acids (DHETs) by sEH [5]. Recent efforts have attempted to develop an sEH inhibitor for the treatment of cardiovascular disease. A previous study found that urea-type inhibitors, such as AUDA and *t*-AUCB, exhibit inhibitory activity on sEH within the nanomolar range [5,11]. However, some limitations of these inhibitors include their low solubility and increased metabolism [12]. The research described herein focuses on efforts to develop a new sEH inhibitor from *S. tamariscina*. The inhibitory activity of isolated selaginellin derivatives **1**–**3** on sEH was examined through enzyme kinetics and docking simulations to provide insight into the interaction between receptor and ligand, as well as a quantitative analysis by comparison with methanol extracts from both the whole plants and roots.

## 2. Results and Discussion

### 2.1. Isolation and Structural Elucidation

**Figure 1:**
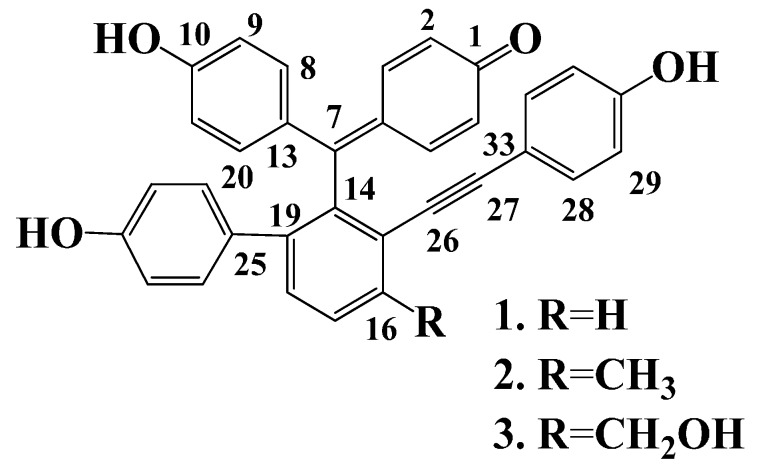
Structures of selaginellin derivatives from *S. tamariscina*.

Whole plants of *S. tamariscina* were extracted with 95% methanol. The extraction was partitioned successively with *n*-hexane, CHCl_3_, EtOAc, and *n*-BuOH. The EtOAc layer was subjected to repeated column chromatography using a silica gel and RP-18 separation to yield known compounds **1**–**3**. These were identified as selaginellin A (**1**) [13], selaginellin B (**2**) [1,13], and selaginellin (**3**) [1,14], by comparison of their spectroscopic data and those reported previously (Figure 1).

### 2.2. Enzyme Inhibition Activity

**Figure 2:**
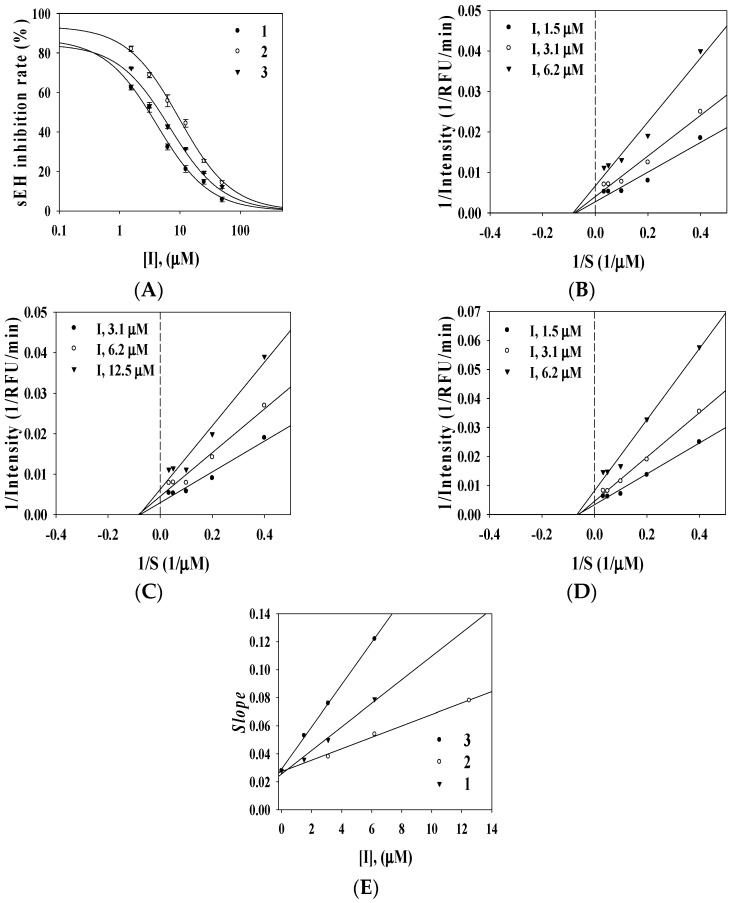
Effects of selaginellin derivatives **1**–**3** on the sEH activity (**A**); Lineweaver-Burk plots of sEH inhibition by **1**–**3** on the hydrolytic activity of sEH (**B**–**D**); and Secondary re-plot of *Slope vs.* [I] (**E**).

To evaluate the inhibitory activity of compounds **1**–**3** on sEH, their hydrolysis effects on the formation of 6-methoxy-2-naphthaldehyde from (3-phenyl-oxiranyl)-acetic acid cyano-(6-methoxy-naphthalen-2-yl)-methyl ester (PHOME) were evaluated by fluorometric determination using an excitation wavelength of 330 nm and emission wavelength of 465 nm [15,16]. AUDA was served as a positive control (IC_50_ 8.2 ± 1.3 nM). They exhibited inhibitory ratios ranges of 92.5 ± 0.8, 89.3 ± 2.1, and 94.5% ± 5.0% of the control value at 100 μM but showed lower than 41% inhibitory ratio on Acetycholinesterase and tyrosinase (Equation (1)). Compounds were confirmed to operate as selective inhibitor on sEH. Furthermore, The results showed that selaginellin A (**1**), selaginellin B (**2**), and selaginellin (**3**) exhibited strong inhibitory activity on sEH in a dose-dependent manner, with IC_50_ values of 3.1 ± 0.1, 8.2 ± 2.2, and 4.2 ± 0.2 μM, respectively (Figure 2A, Table 1). To evaluate the binding mode between sEH and the inhibitors, a kinetic study was performed using various inhibitors and substrate concentrations. The respective Lineweaver-Burk plots (Equation (2)) indicated that all compounds (**1**–**3**) were non-competitive inhibitors of sEH (Figure 2B–D), represented by a family of straight lines with varying slopes with a joint intercept on the abscissa. Using Equation (3), the *Ki* values were calculated to be 2.9 ± 1.2, 6.8 ± 0.5, and 1.8 ± 1.5 μM, respectively (Figure 2E, Table 1).

**Table 1:** Inhibitory effects of selaginellin derivatives (**1**–**3**) on sEH activity.

Compound	Inhibitory Rate at 100 μM (%) ^a^			sEH ^a^	
	AChE	Tyrosinase	sEH	IC_50_ (μM)	Type (*Ki*, μM)
**1**	32.8 ± 2.1	36.2 ± 2.1	92.5 ± 0.8	3.1 ± 0.1	Noncompetitive (2.9 ± 1.2)
**2**	19.6 ± 1.0	32.5 ± 1.5	89.3 ± 2.1	8.2 ± 2.2	Noncompetitive (6.8 ± 0.5)
**3**	12.2 ± 0.8	40.1 ± 3.2	94.5 ± 5.0	4.2 ± 0.2	Noncompetitive (1.8 ± 1.5)
Positive control ^b^	1.4 ± 3.5 μM	25.2 ± 1.2 μM	8.2 ± 1.3 nM		

^a^ All compounds examined in a set of triplicated experiments; ^b^ Positive control. (AChE: Galantamine; tyrosinase: kojic acid; sEH: AUDA).

### 2.3. Docking Calculation

**Figure 3:**
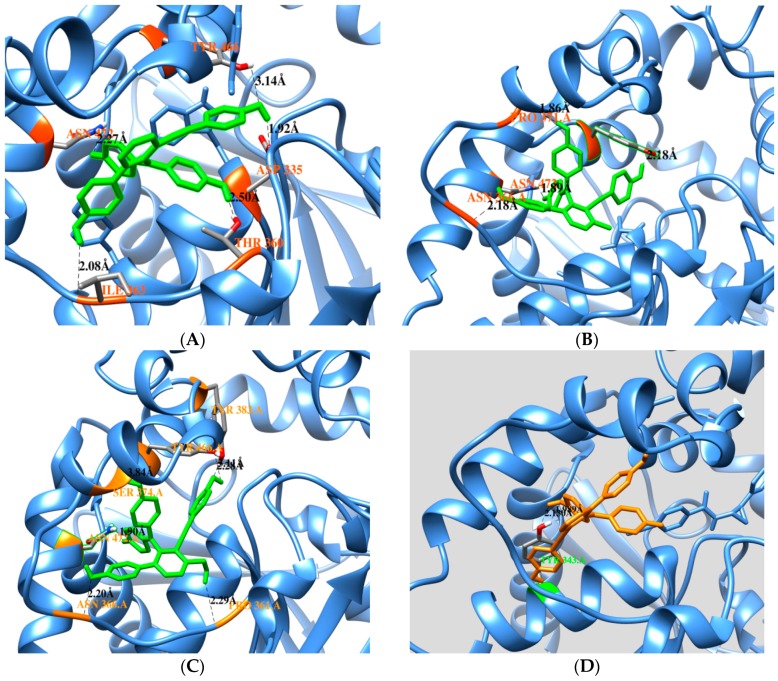

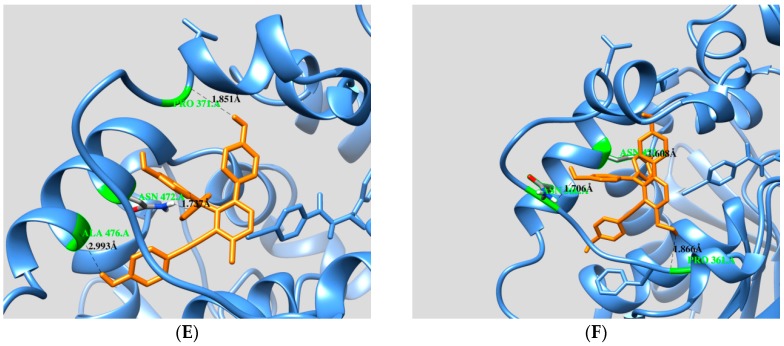
Binding pose at the lowest energy between compounds **1**–**3** and free enzyme (**A**–**C**); binding pose at the lowest energy between compounds for **1**–**3** and enzyme-substrate complex (**D**–**F**).

For insight into the interaction between molecule and ligand, a docking simulation was performed using the AutoDock software (version 4.2) from The Scripps Research Institute [16,17]. On the basis of a non-competitive mechanism, the docking simulation was tested in the blind docking of each condition on the free enzyme or enzyme-substrate complex (PDB ID: 3ANS from RCSB) with the three-dimensional structure of the ligand, which was sketched and structurally optimized by minimization with minimun RMS gradient (0.01) implemented in Chem3D 12.0 (ChembridgeSoft). The predicted binding site was selected so that the favorable cluster possessed the lowest energy and the largest number of superpositions of the ligand on the enzyme. A cluster analysis was performed by 2.0 Å rmsd from 50 docking runs (Supporting information, Figure S11). Figure 3A–F displays the interactions between selaginellin A (**1**), selaginellin B (**2**), and selaginellin (**3**) with the free enzyme and the enzyme-substrate complex. The ligand from the original protein database was suggested as a substrate due to the binding form in the active site. These results revealed differences in the predicted binding sites of **1**–**3** in two situations: the docked pose of **1** with free enzyme (binding energy −10.81 kcal/mol) showed that the three hydroxyl groups and one ketone group were bound by hydrogen to ASN472 (2.2 Å), ILE363 (2.0 Å), THR360 (2.5 Å), ASP335 (1.9 Å), and TYR466 (3.1 Å). Moreover, **2** and **3** appeared in a similar position, with binding energies equal to −10.76 and −10.24 kcal/mol, respectively. Compound **2** also interacted with residues ASN366 (2.1 Å), ASN472 (1.8 Å), PRO371 (1.8 Å), and TYR446 (2.1 Å). Compound **3** interacted via only hydrogen bonding with residues ASN366 (2.2 Å), ASN472 (1.8 Å), TYR383 (2.2 Å), PRO361 (2.2 Å), and TYR 466 (2.4/3.1 Å). In a docking simulation with the enzyme-substrate complex, compound **1** (−9.45 kcal/mol) showed that a hydroxyl group established hydrogen bonds with the side chains of TYR343 (1.9/2.1 Å). Compound **2** (−11.8 kcal/mol) was involved in hydrogen bonding with ASN472 (1.7 Å), PRO371 (1.9 Å), and ALA476 (2.9 Å). Analysis of **3** (−10.87 kcal/mol) showed hydrogen-bonding interactions with ASN452 (1.6 Å), PRO361 (1.8 Å), and ASN366 (1.7 Å) (Table 2). These bound to predicted binding sites including a portion of the active site, with free enzyme exhibiting the best pose at pocket A, which was located far from the active site within the enzyme-substrate complex (Figure S12).

**Table 2:** Docking interactions of inhibitors against sEH.

**Free Enzyme**	**Affinity (kcal/mol)**	**Residues in Close Contact**	**Hydrogen Bond (Å)**
Selaginelin A	−10.81	Asp335, Trp336, Met339, Thr360, Ile363, Phe381, Tyr383, Gln384, Tyr466,Met469, Asn472, Val498, Leu499	Asn472(2.2), Ile363(2.0), Thr360(2.5), Asp335(1.9), Tyr466(3.1)
Selaginelin B	−10.76	Trp336, Met339, Tyr343, Thr360, Pro361, Ile363, Asn366, Pro371, Asn472, Ser374, Tyr466, Met469, Leu499, Met503	Asn366(2.1), Asn372(1.8), Pro371(1.8), Typ466(2.1)
selaginelin	−10.24	Trp336, Met339, Thr360, Pro361, Ile363, Asn366, Met369, Pro371, Ser374, Ile375, Tyr383, Gln384, Tyr466, Met469, Asn472, Trp473, Leu499, Met503	Asn366(2.2), Asn472(1.8), Tyr383(2.2), Pro361(2.2), Tyr466 (2.4,3.1)
**Enzyme-Substrate Complex**	**Affinity (kcal/mol)**	**Residues in Close Contact**	**Hydrogen Bond (Å)**
Selaginelin A	−9.45	Met339, Tyr343, Phe362, Ile363, Asn472, Ser374, Phe381, Ala476, Met503	Tyr343(2.1,1.9)
Selaginelin B	−11.8	Trp336, Met339, Tyr343, Leu346, Pro361, Phe362, Ile363, Ala365, Asn366, Met369, Pro371Ile375, Gln384, Met469, Asn472, Tpr473, Ala476,	Ala476(2.9), Asn 472(1.7), Pro371(1.8)
selaginelin	−10.87	Trp336, Met339, Tyr343, Leu346, Pro361, Phe362, Ile363, Asn366, Met369, Pro371, Ser374, Ile375, Gln384, Met469, Asn472, Trp473, Ala476	Asn366(1.7), Asn452(1.6), Pro361(1.8)

### 2.4. HPLC Analysis

**Figure 4:**
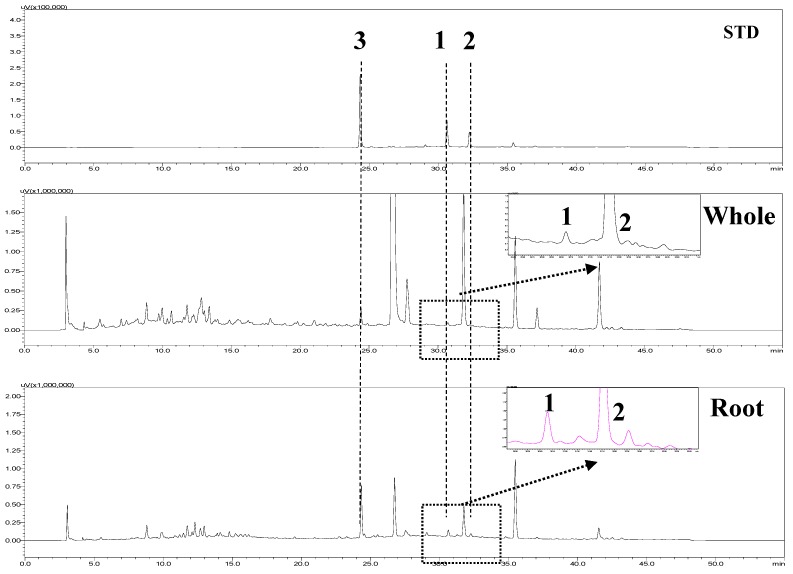
Pattern analysis of selaginellin A (**1**), selaginellin B (**2**), and selaginellin (**3**) in methanol extracts (0.5 mg/mL) from the *S. tamariscina* whole plants and roots.

High-performance liquid chromatography (HPLC) was used to separate selaginellin derivatives **1**–**3** and compare the quantitative content between methanol extracts of whole plant and roots of *S. tamariscina.* Mixed standards (**1**–**3**) were measured by HPLC at 280 nm (Figure 4). Peak retention times were as follows: compound **3**, Rt = 24.6 min; compound **1**, Rt = 31.0 min; compound **2**, Rt = 32.6 min. Profiles of extracts **1**–**3** were confirmed by comparing their peak retention times with those of the standards (Figure 4). Their contents were calculated as shown in Table 3. The selaginellin B (**2**) and selaginellin (**3**) contents in the whole *S. tamariscina* plant were similar to those reported previously [18]. The contents of the individual compounds differed markedly depending on whether they were extracted from the whole plants or roots. The contents of **1**–**3** were low in whole methanol extracts. The total amount of selaginellin **3** was three- and six-fold higher than that of selaginellin A (**1**) and B (**2**), respectively. However, the contents of **1**–**3** from root extracts were three- to four-fold higher than those from whole methanol extracts. Specifically, selaginellin **3** was confirmed, with a content of 189.3 ± 0.0 μg/g in the dried roots of *S. tamariscina*, which was revaluated as a high precious part to obtain selaginellin **3** (Table 3). The coefficient of correlation (R^2^) > 0.99 of the three standards indicated good linearity. Limits of detection (LODs) of the method ranged from 0.1–0.2 μg/mL, and limits of quantitation (LOQs) ranged from 0.3–0.6 μg/mL (Table S1). Furthermore, the precision values (% relative standard deviation [RSD]) were 0.3%–2.1% for intra-day and 0.5%–1.6% for inter-day. However, the accuracy (%) was 98.2%–102.5% for intra-day and 96.1%–105.4% for inter-day. Thus, the newly developed method was validated in terms of its LOD, LOQ, linearity, precision (intra- and inter-day), and accuracy (intra- and inter-day) (Table S2).

**Table 3:** Content of the analyzed selaginellins (**1**–**3**) in methanol extracts from each part of *S. tamariscina.*

Parts	Content (μg/g, Dried Material, *n* = 3)		
	Selaginellin	Selaginellin A	Selaginellin B
Whole	61.2 ± 0.3 ^a^	22.9 ± 0.1	10.2 ± 0.3
Root	189.3 ± 0.0	56.0 ± 0.1	42.5 ± 0.1

^a^ Data are expressed as Mean ± S.D.

## 3. Materials and Methods

### 3.1. General Experimental Procedures

NMR spectra was obtained on Bruker Advance 600 and 300 MHz (Bruker; Rheinstetten, Germany) spectrometer. Column chromatography was performed using silica gel (Kieselgel 60; 70–230; and 230–400 mesh; Merck; Darmstadt, Germany) and YMC RP-18 resins. Thin layer chromatography (TLC) was performed using pre-coated silica-gel 60 F_254_ and RP-18 F_254S_ plates (both 0.25 mm; Merck; Darmstadt, Germany). Spots were visualized by spraying with 10% aqueous H_2_SO_4_ solution followed by heating. Methanol; acetonitrile (Sigma-Aldrich; St. Louis, MO, USA) and trifluoroacetic acid (Alfa Aesar; Ward Hill, MA, USA) used in this study were of HPLC grade and the distilled water was prepared by the ultra-pure water manufacturing device (Optimos; Sinhan Science Tech, Daejeon, Korea). The chromatographic system was Shimazdu prominence system equipped with a SPD-20A dual UV-Vis detector and installed with a Shimadzu LC solution software (Ver. 1.25; Shimadzu; Kyoto, Japan). The quantitative analysis for three selaginellins was carried out on Optimapak C18 (250 mm × 4.6 mm I.D.; 5 μm) column. Soluble epoxide hydrolases (human recombinant, 10011669) and PHOME (10009134) were purchased from Cayman (Ann Arbor, MI, USA).

### 3.2. Plant Material

*S. tamariscina* was purchased from an herbal market at Kumsan, Chungnam, Korea, in December 2013. This species was identified by Prof. Y. H. Kim. A voucher specimen (CNI-13106) was deposited at the herbarium, College of Pharmacy, Chungnam National University (CNU).

### 3.3. Extraction and Isolation

Dried whole plant (2.4 kg) was extracted three times with 95% methanol (36 L) at 50 °C for 5 h. Concentrated methanol extract (270 g) was suspended in distilled water (2.5 L) and successively partitioned with *n*-hexane, chloroform, ethyl acetate, and buthanol. Ethyl acetate layer (21 g) was chromatographed with silica gel column chromatography using the gradient system of chloroform and methanol (from 100:1 to 1:1) to obtain seven fractions (2A to 2G). Fraction 2D was subjected to silica gel column chromatography with gradient solvent system of chloroform and methanol (from 50:1 to 0.5:1) to achieve eight fractions (2DA to 2DH). Fraction 2DF was divided by RP-18 column chromatography using the isocratic solvent system of 60% methanol, and then pure compound, selaginellin (**3**) was gained. Fraction 2DE was chromatographed with RP-18 column chromatography using the gradient solvent system of distilled water and methanol (from 100:1 to 0.2:1) to achieve selaginellin A (**1**) and B (**2**).

### 3.4. Enzymatic Assay

Briefly, 130 μL of sEH (62.5 ng/mL) in 25 mM bis-Tris-HCl buffer (pH 7.0) containing 0.1% BSA and 20 μL of various concentrations of the inhibitors dissolved in MeOH were added in a 96 well plate and then mixed with 50 μL of 40 μM PHOME at 37 °C. The mixture was recorded at excitation and emission of 330 and 465 nm for one hour [15]. The inhibitory ratio was calculated according to the following equation:

Inhibitory activity rate (%) = 100 − [(*S*_60_ − *S*_0_)/(*C*_60_ − *C*_0_)] × 100 (1)

where *C*_60_ and *S*_60_ were the fluorescence of control and inhibitor after 60 min, *S*_0_ and C_0_ were the fluorescence of control and inhibitor at zero min.

### 3.5. sEH Kinetic Analysis

To analyze the non-competitive inhibition, the Lineweaver-Burk equation can be written in double reciprocal form:

(2) $$\frac{1}{V}=\frac{Km}{V\max}\left(1+\frac{\left[I\right]}{Ki}\right)\frac{1}{\left[S\right]}+\frac{1}{V\max}\left(1+\frac{\left[I\right]}{Ki}\right)$$

Secondary plots can be constructed from

(3) $$Slope=\frac{km}{V\max}+\frac{Km[I]}{V\max Ki}$$

### 3.6. Docking Calculation

The 3D structure of the ligand was modeled and minimized by MM2 using the Chem3D Pro (version: 12.0). The flexible bonds of ligand were assigned with AutoDockTools. The protein structure (PDB ID: 3ANS) was downloaded from RCSB (protein data bank), after which the water, or the water and inhibitor suggested as substrate were removed from receptor complexes using Chimera. All hydrogen atoms and gasteiger charges in two protein structures were added. Simulation studies were performed using the AutoDock program (version 4.2). Briefly, to perform blind docking in AutoDock 4.2, the grid dimensions were established using grid enzyme center, number of points (X: 126, Y: 126, Z: 126), and spacing (0.375 Å). Docking simulation of protein structures and ligand were performed using the Lamarckian Genetic Algorithm (Runs 50 and the maximum number of evaluations was set as long). The docking simulation results were prepared using Chimera [16,17].

### 3.7. HPLC Analysis Condition

The quantitative analysis for three selaginellin derivatives **1**–**3** from each sample solution was performed on a Shimadzu Prominence system. All samples were analyzed on an Optimapak ODS C18 column by mobile phase constituted with (A) 0.1% trifluoroacetic acid aqueous solution and (B) acetonitrile containing with 0.1% trifluoroacetic acid. The elution conditions applied were: 5%–75% B for 40 min, 75% B for 5 min, 75%–5% B for 1 min, and 5% B for 9 min, successively. The flow rate and injection volume were 1 mL/min and 20 μL, respectively. This system was operated at 30 °C. All peaks were detected at wavelength of 280 nm. Finally, total run time of quantitative analysis was 55 min.

### 3.8. Statistical Analysis

All activity tests in the presence of inhibitors were performed in triplicate and results are presented as the means ± standard error of the mean (SEM). The results were subjected to analysis using Sigma Plot (SPP Inc., Chicago, IL, USA).

## 4. Conclusions

Selaginellin derivatives (**1**–**3**) generated with a rare carbon scaffold were isolated from *S. tamariscina* using silica gel, C-18, and Sephadex LH-20 column chromatography. The derivatives exhibited low IC_50_ values of 3.1 ± 0.1 (**1**), 8.2 ± 2.2 (**2**), and 4.2 ± 0.2 μM (**3**) against sEH. Regarding enzyme kinetics, Lineweaver-Burk plots indicated that lines generating using various inhibitor concentrations crossed the equal x-intercept on the abscissa, and Equation (3) of secondary plots yielded the following *Ki* values: **1**, 2.9 ± 1.2; **2**, 6.8 ± 0.5; **3**, 1.8 ± 1.5 μM. The derivatives were confirmed to interact with sEH as non-competitive inhibitors. Therefore, the enzyme is involved in the reaction in two of four states: free enzyme, enzyme-substrate complex, enzyme-inhibitor complex, or enzyme-substrate-inhibitor complex. Selaginellin derivatives **1**–**3** likely bind with the enzyme via two routes, the free enzyme or enzyme-substrate complex. Therefore, compounds **1**–**3** were subjected to a docking simulation with free enzyme and the enzyme-substrate complex, respectively. In the former route, all derivatives occupied pocket A and a small portion of the substrate in the free enzyme, but only pocket A of the enzyme-substrate complex in the latter route. This study suggests that compounds **1**–**3** occupy pocket A and a small portion of the substrate when bound to free enzymes. Furthermore, when the substrate was added to the active site, the compounds could fully slide into pocket A simultaneously. Regarding the second route, **1**–**3** may directly bind to pocket A when bound to the enzyme-substrate complex. Additionally, according to these docking simulation results, ASN 472 in pocket A, which is the positively charged amino acid, plays the common catalytic residues relating to the binding with all selaginellin derivatives (**1**–**3**). These proposals may be a clue for the development of new sEH inhibitor instead of urea-type inhibitors. Furthermore, an HPLC analysis of selaginellin derivative (**1**–**3**) contents in whole plants and roots of *S. tamariscina* was developed and validated for the first time. The contents of compounds **1**–**3** were higher in the root extract than in whole plants. Among them, selaginellin **3** exhibited the highest content in dried roots (189.3 ± 0.0 μg per 1 g). This study was the first to analyze selaginellins in the roots and whole plants of *S. tamariscina*, and included evaluation of their sEH inhibitory activity, enzyme kinetics, and docking simulations. Overall, we conclude that selaginellin derivatives **1**–**3** are a promising lead compounds for development of inhibitor targeting sEH.

## Supplementary Materials

1D/2D-NMR and ESI-MS spectra, Docking pose, and validation on **1**–**3** by HPLC. Supplementary materials can be accessed at: http://www.mdpi.com/1420-3049/20/12/19774/s1.
