# Supplementary material for: Soluble Epoxide Hydrolase Inhibitory Activity of Selaginellin Derivatives from Selaginella tamariscina
Source: Molecules. 2015 Dec 2;20(12):21405–14. doi: 10.3390/molecules201219774 (PMC6331899; doi:10.3390/molecules201219774)
Supplement: Supplementary file 1 [file molecules-20-19774-s001.pdf]

Supplementary Materials: Soluble Epoxide Hydrolase Inhibitory Activity of Selaginellin Derivatives from *Selaginella tamariscina*

Jang Hoon Kim, Chong Woon Cho, Bui Huu Tai, Seo Young Yang, Gug-seoun Choi, Jong Seong Kang and Young Ho Kim

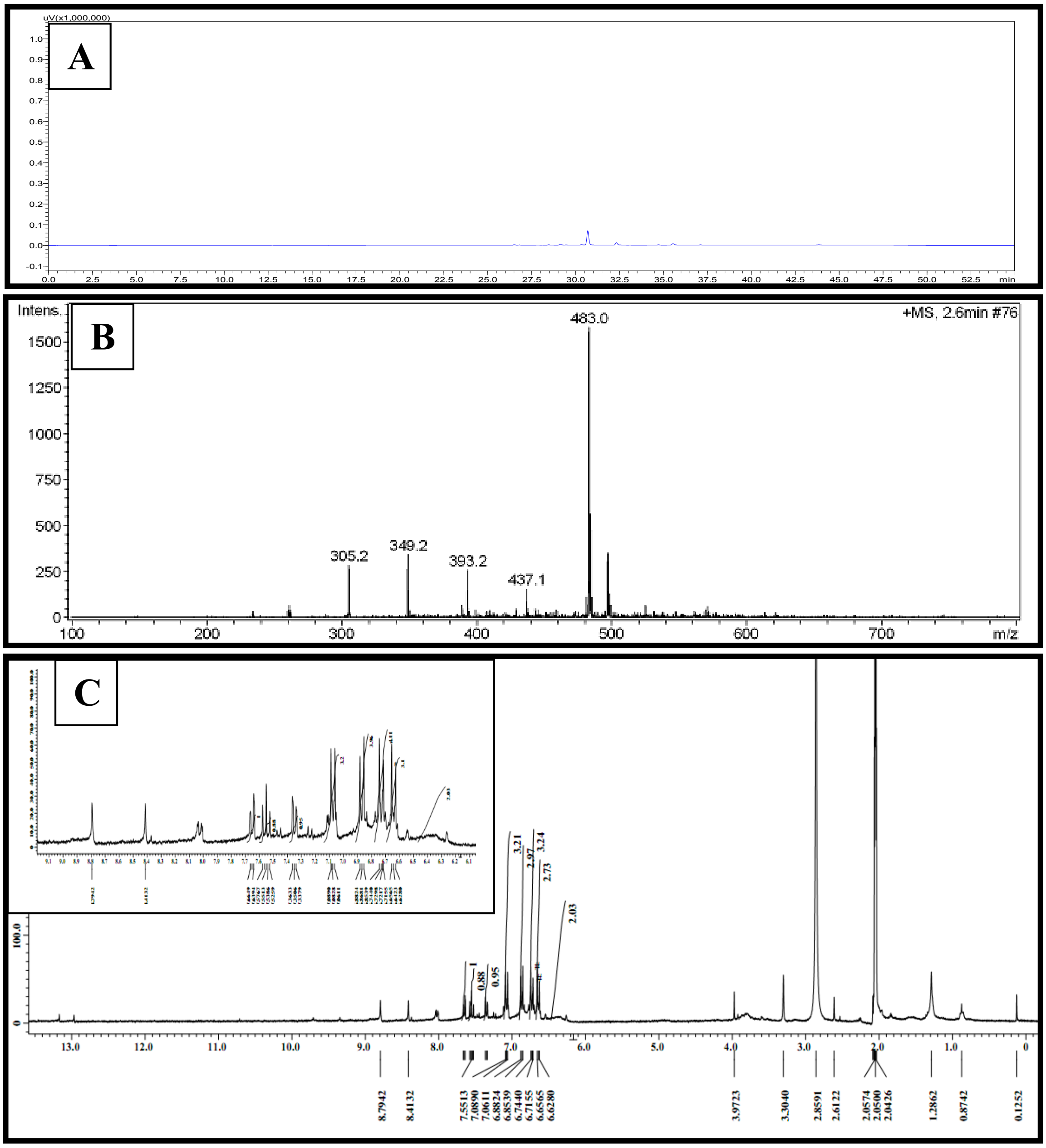

Figure S1. HPLC (**A**); ESI-MS (**B**); and ^{1}H-NMR spectra (**C**) of compound **1** (300 MHz, $CDCl_3$).

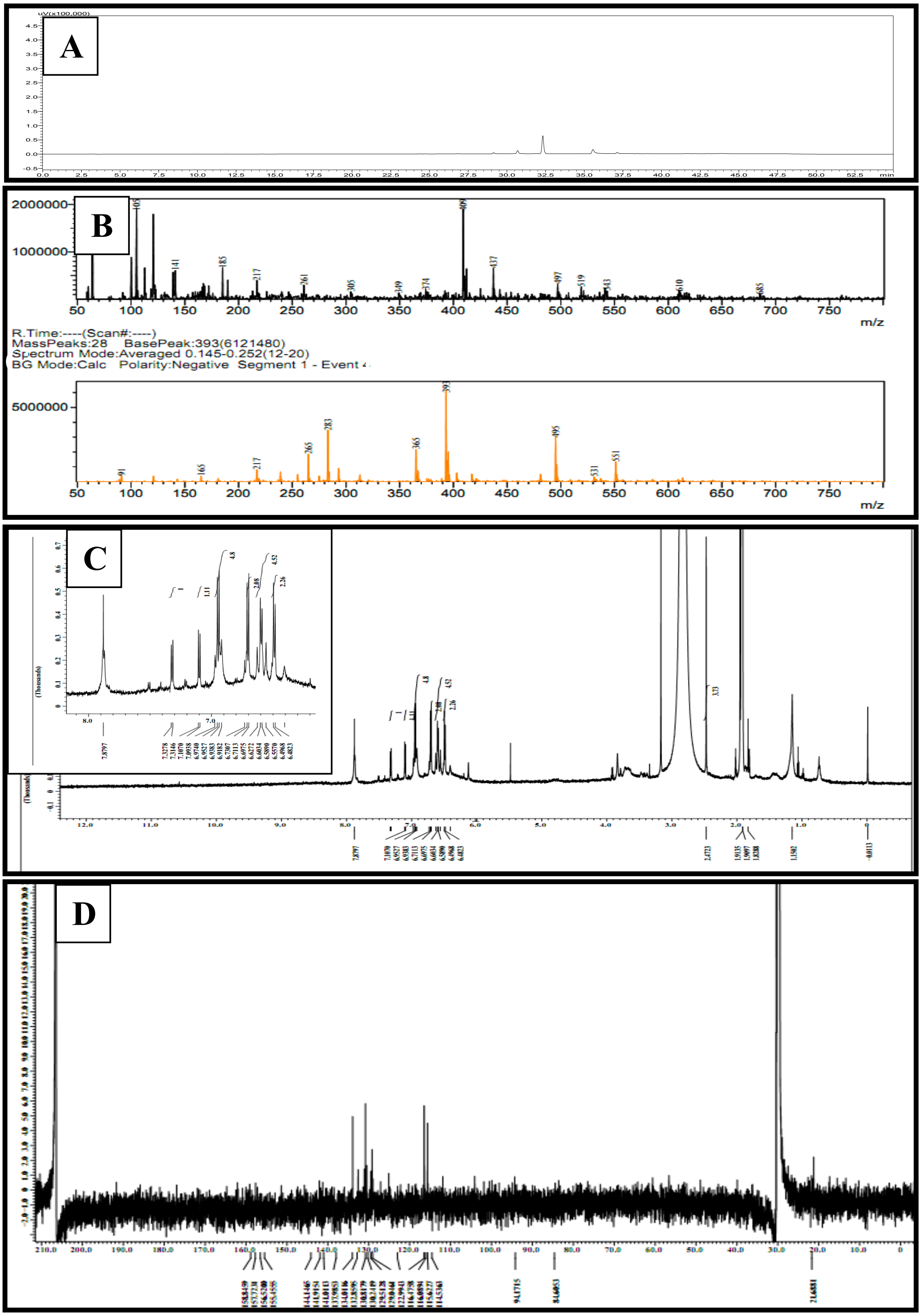

Figure S2. HPLC (**A**); ESI-MS (**B**); ^{1}H-NMR spectra (**C**) and ^{13}C-NMR (**D**) spectra of compound **2** (600 MHz, $CDCl_3$).

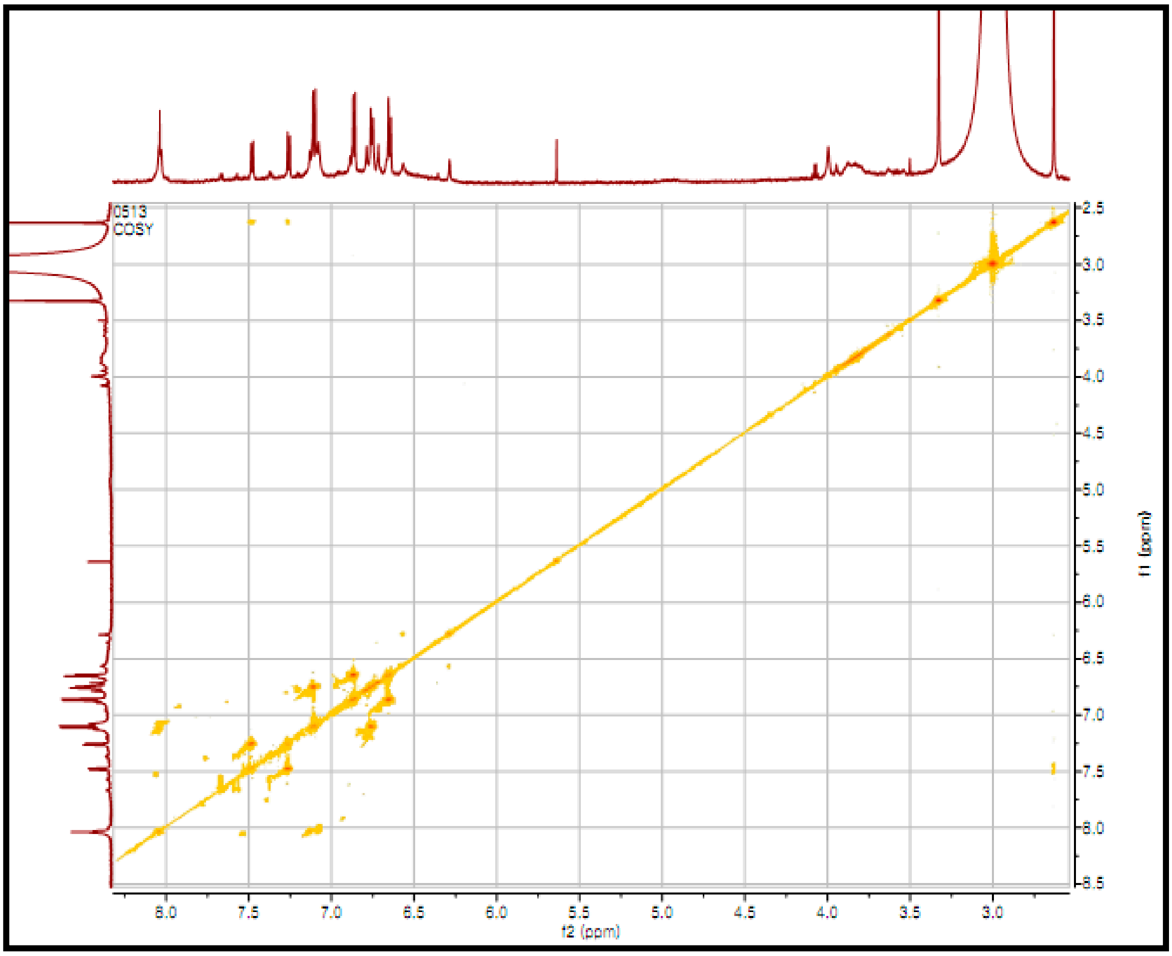

Figure S3. COSY spectrum of compound **2**.

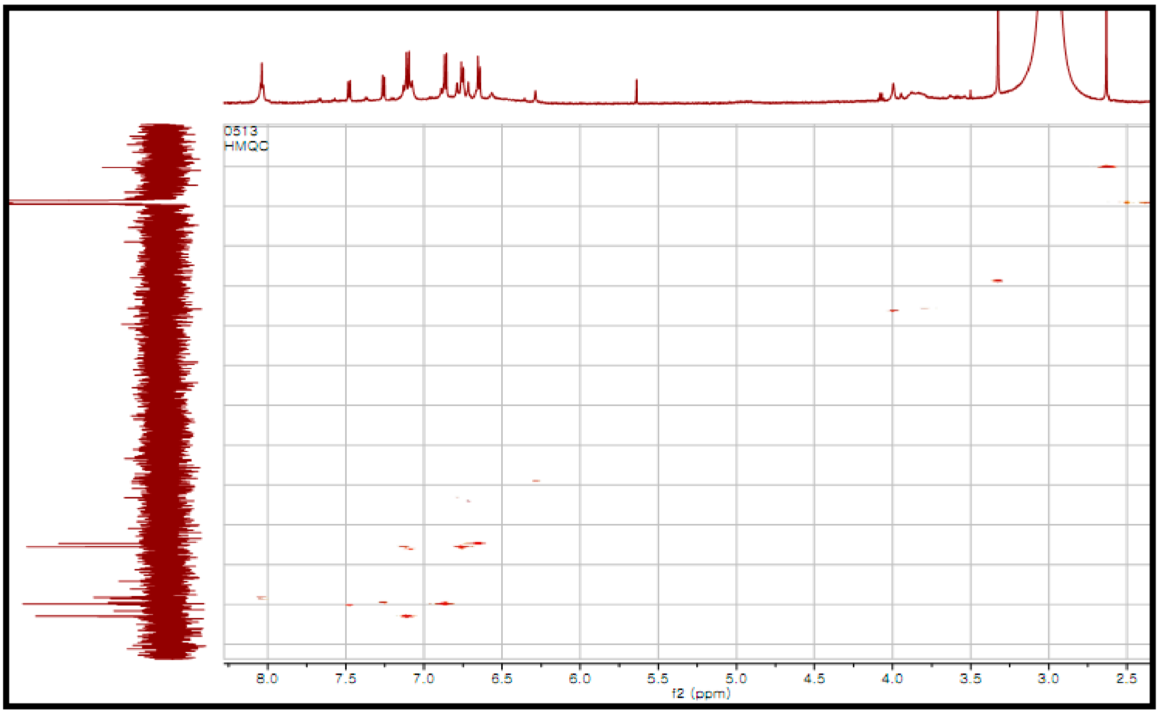

Figure S4. HMQC spectrum of compound **2**.

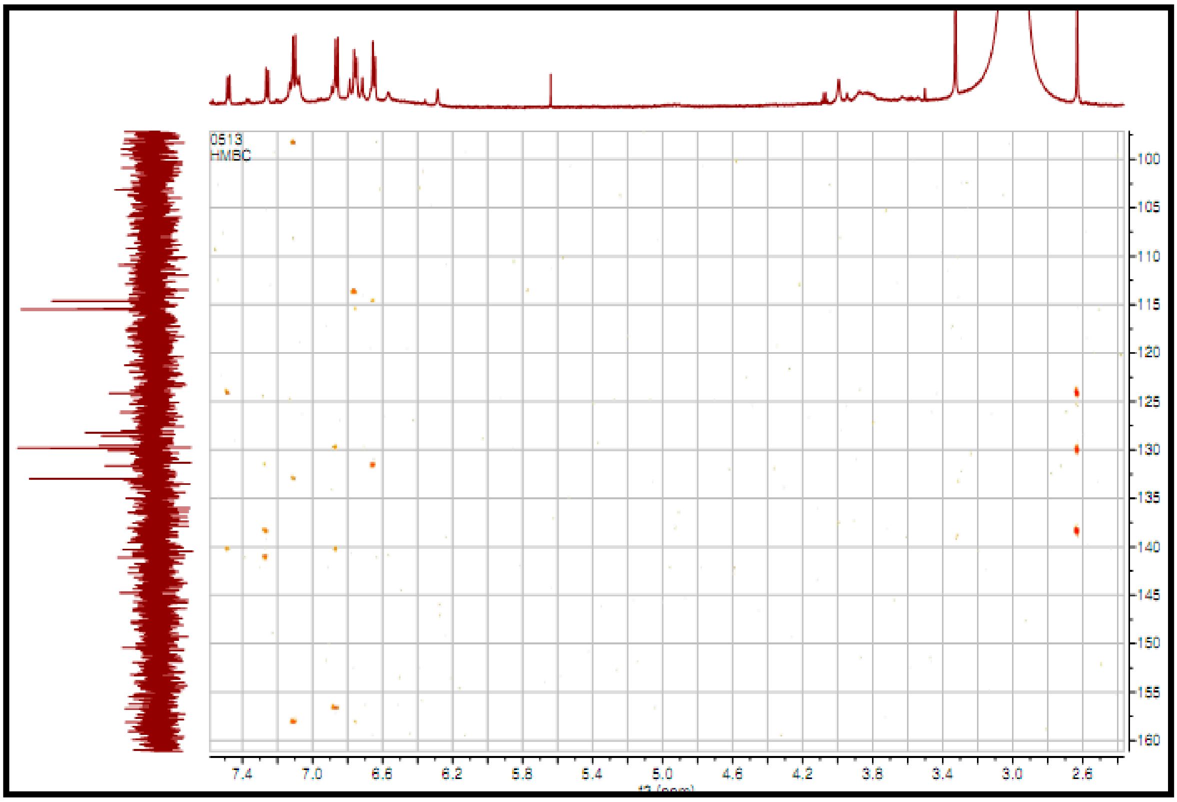

Figure S5. HMBC spectrum of compound **2**.

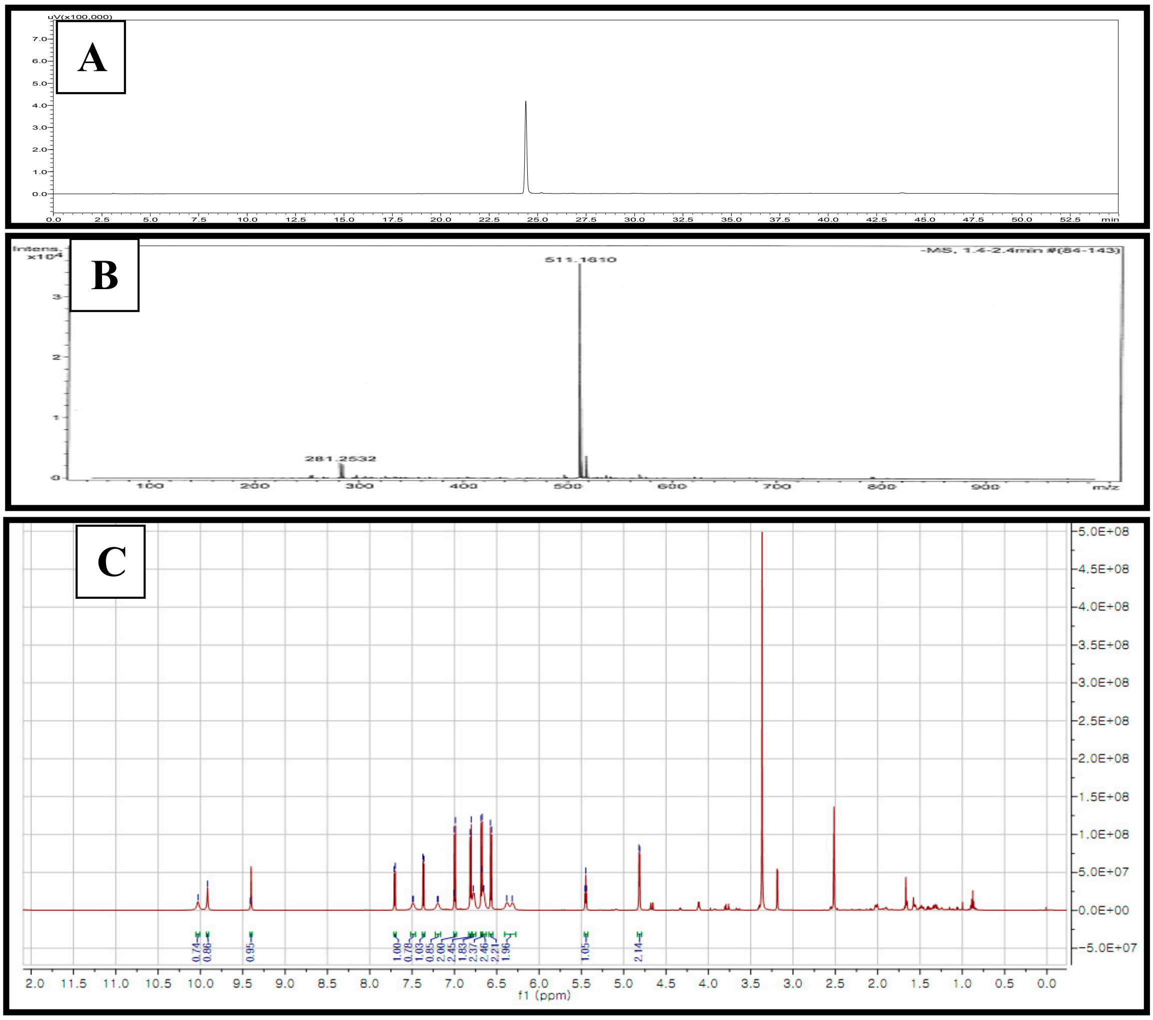

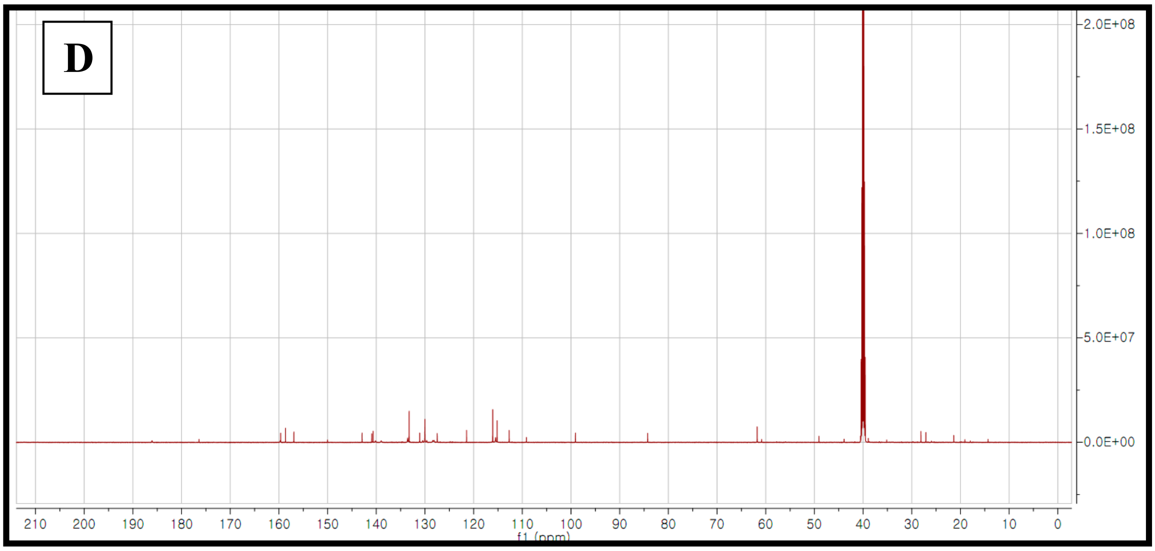

Figure S6. HPLC (**A**); ESI-MS (**B**); ^{1}H-NMR spectra (**C**) and ^{13}C-NMR (**D**) spectra of compound **3** (600 MHz, $CDCl_3$).

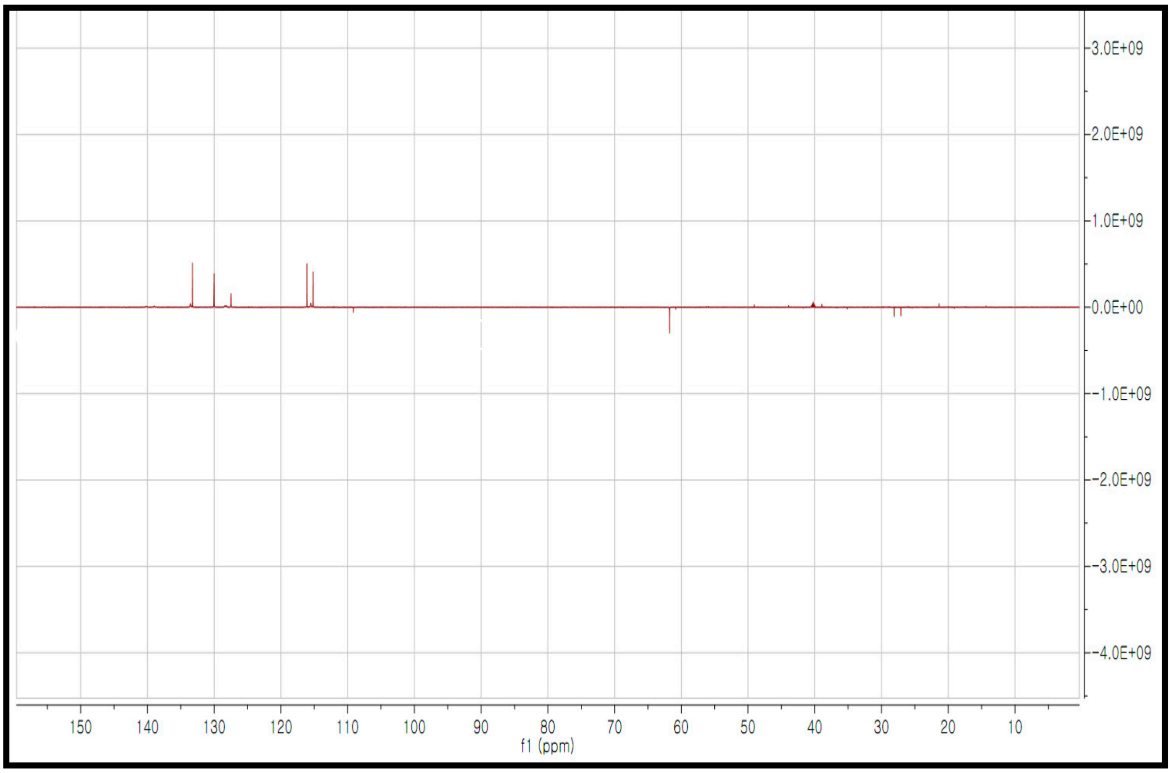

Figure S7. DEPT135° spectrum of compound **3**.

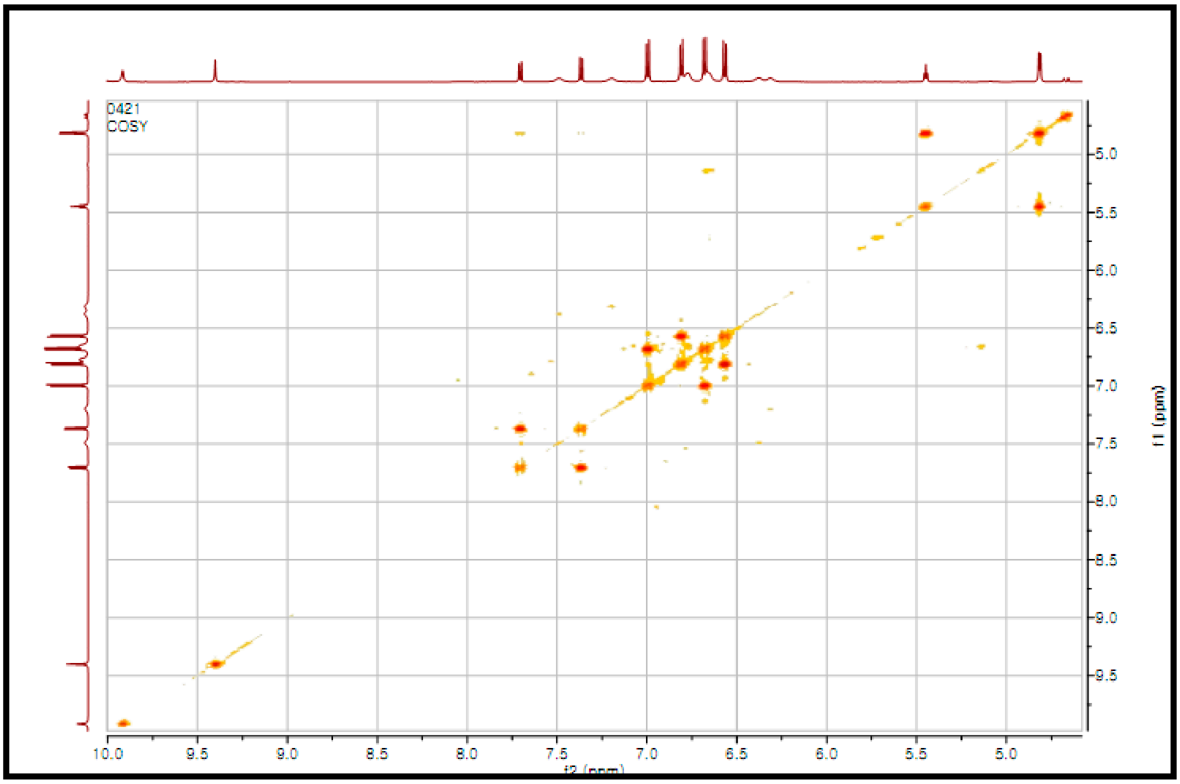

Figure S8. COSY spectrum of compound **3**.

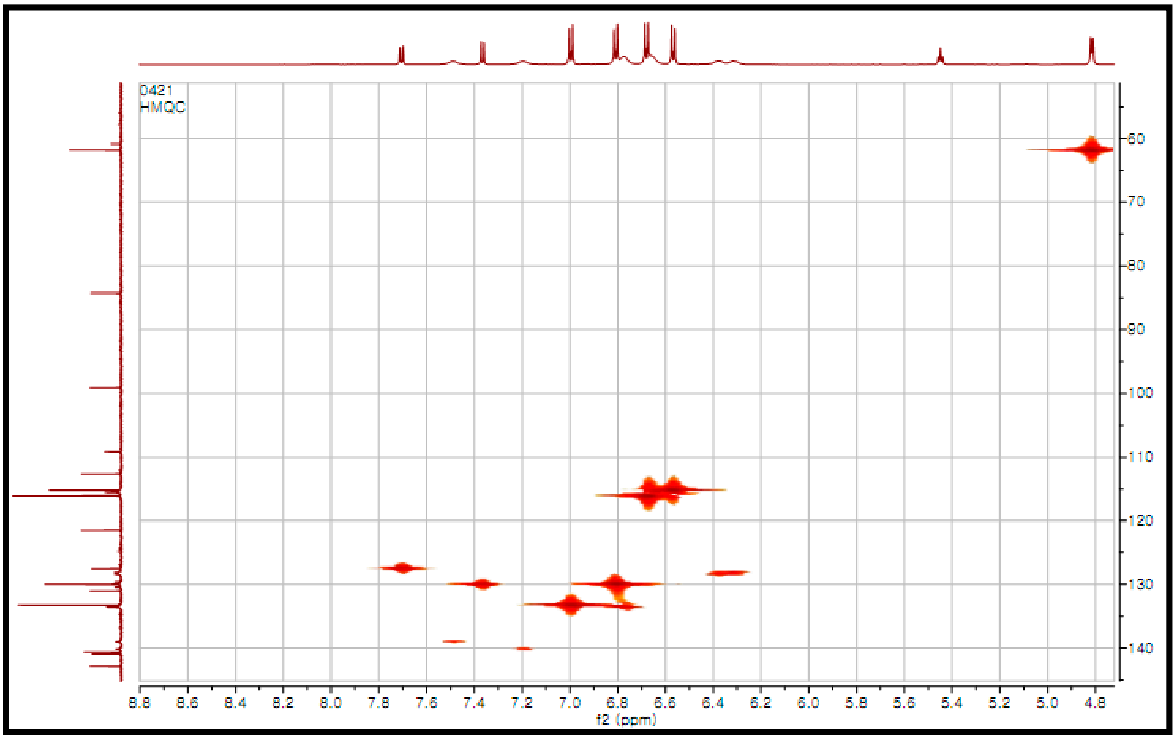

Figure S9. HMQC spectrum of compound **3**.

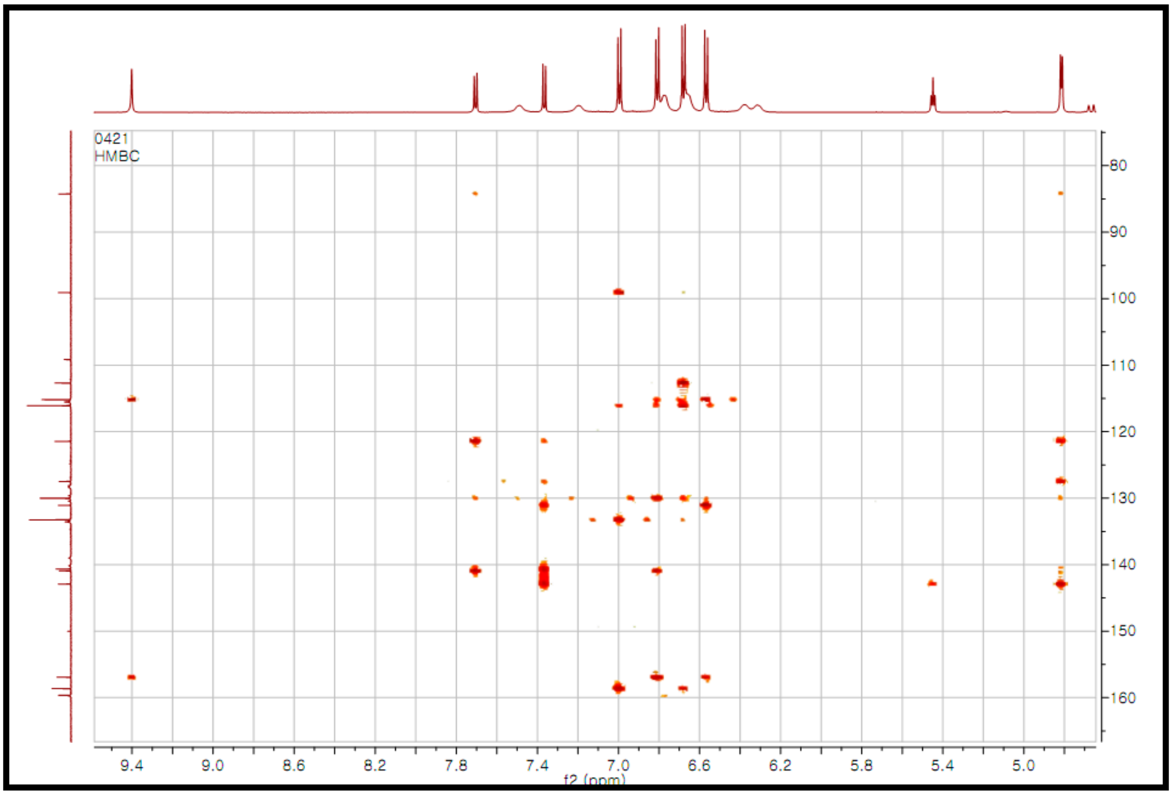

Figure S10. HMQC spectrum of compound **3**.

selaginellin A (**1**) Red powder; mp 180–185 °C; ESI-MS *m/z* = 483.2 [M + H]$^+$; ^{1}H-NMR (400 MHz, Acetone-*d*$_5$) δ 7.65 (d, *J* = 7.6 Hz, 1H, H-16), 7.55 (t, *J* = 7.6 Hz, 1H, H-16 = 7), 7.36 (d, *J* = 7.6 Hz, 1H, H-18), 7.08 (d, *J* = 8.3 Hz, 2H, H-28,32), 7.07 (d, *J* = 7.6 Hz, 2H, H-3,5), 6.86 (d, *J* = 8.3 Hz, 2H, H-20,24), 6.72 (d, *J* = 8.3 Hz, 2H, H-29,31), 6.64 (s, 2H, H-9,11,21,23), 6.35 (s, 2H, H-2,6); ^{13}C-NMR (100 MHz, Acetone-*d*$_5$) δ 186.8 (C-1), 159.0 (C-7,10,30), 158.7 (C-22), 144.0 (C-19), 142.0 (C-14), 140.5 (C-3), 139.2 (C-5), 134.0 (C-13), 133.9 (C-8,12), 132.6 (C-25), 131.7 (C-28,32), 131.1 (C-18), 131.0 (C-20,24), 130.9 (C-17), 130.0 (C-4), 129.5 (C-2,6), 125.6 (C-15), 116.4 (C-29,31), 115.6 (C-9,11,21,23), 114.5 (C-33), 94.4 (C-27), 87.5 (C-26).

selaginellin B (**2**) Red powder; mp 192–195 °C; ^{1}H-NMR (600 MHz, Acetone-*d*$_5$) δ 7.32 (d, *J* = 8.5 Hz, 1H, H-17), 7.10 (d, *J* = 8.5 Hz, 1H, H-18), 6.94 (d, *J* = 8.5 Hz, 4H, H-3,5,8,12), 6.71 (d, *J* = 8.5 Hz, 4H, H-20,24,28,32), 6.59 (d, *J* = 8.5 Hz, 4H, H-2,6,9,11), 6.49 (d, *J* = 8.5 Hz, 4H, H-21,23,29,31), 2.47 (s, 3H); ^{13}C-NMR (150 MHz, Acetone-*d*$_5$) δ 158.8 (C-1), 157.7 (C-10,30), 156.5 (C-22), 144.1, 141.9, 140.1, 137.9, 134.0 (C-3, 5), 132.8 (C-17), 130.8 (C-20,24,28,32), 129.5, 122.9, 116.4 (C-2,6,9,11), 116.0, 115.6 (C-21,23,29,31), 94.1 (C-27), 84.6 (C-26), 21.6 (C-34).

selaginellin (**3**) Red powder; mp 191–193 °C; ESI-MS *m/z* = 511.16 [M − H]$^-$; ^{1}H-NMR (600 MHz, DMSO-*d*$_6$) δ 7.70 (d, *J* = 8.5 Hz, 1H, H-17), 7.49 (s, 1H, H-5), 7.36 (d, *J* = 8.5 Hz, 1H, H-18) 7.20 (s, 1H, H-6), 7.00 (d, *J* = 8.5 Hz, 2H, H-28,32), 6.81 (d, *J* = 8.5 Hz, 2H, H-20,24), 6.80 (s, 2H), 6.67 (d, *J* = 8.5 Hz, 2H, H-29,3), 6.65 (s, 2H), 6.57 (d, *J* = 8.5 Hz, 2H, H-21,23), 6.38 (s, 1H, H-2), 632 (s, 1H, H-6), 5.45 (t, *J* = 6.5 Hz, 1H, OH-34), 4.81 (d, *J* = 6.5 Hz, 2H, H-34); ^{13}C-NMR (150 MHz, DMSO-*d*$_6$) δ 159.1 (C-1), 158.1 (C-10, 30), 156.4 (C-22), 142.4 (C-16), 140.4 (C-19), 140.1 (C-18), 139.7 (C-6), 138.5 (C-5), 132.8 (C-8,12), 132.7 (C-28,32), 130.6 (C-25), 129.5 (C-18,20,24), 127.0 (C-2,6,17), 120.9 (C-15), 115.6 (C-29,31), 115.0 (C-9, 11), 114.7 (C-21,23), 112.2 (C-33), 98.6 (C-27), 83.7 (C-26), 61.2 (C-34).

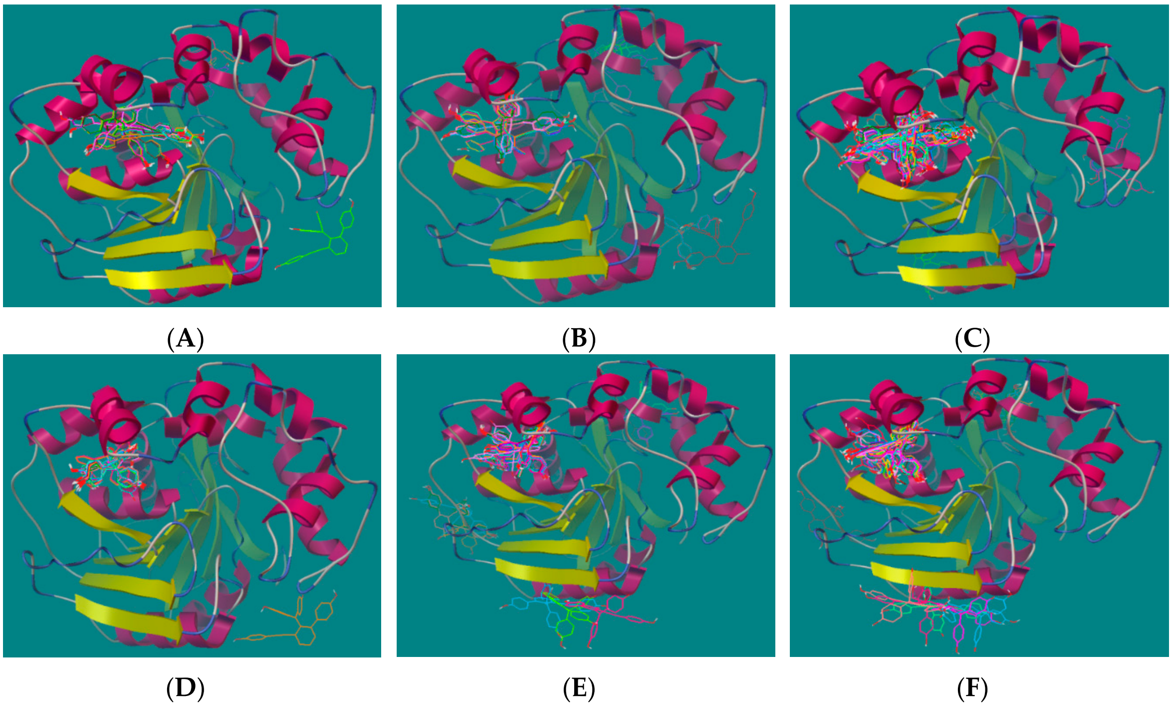

Figure S11. (**A**–**C**) The favorable clusters were shown between **1**–**3** and free enzyme; (**D**–**F**) The favorable clusters were also shown between **1**–**3** with enzyme-substrate complex.

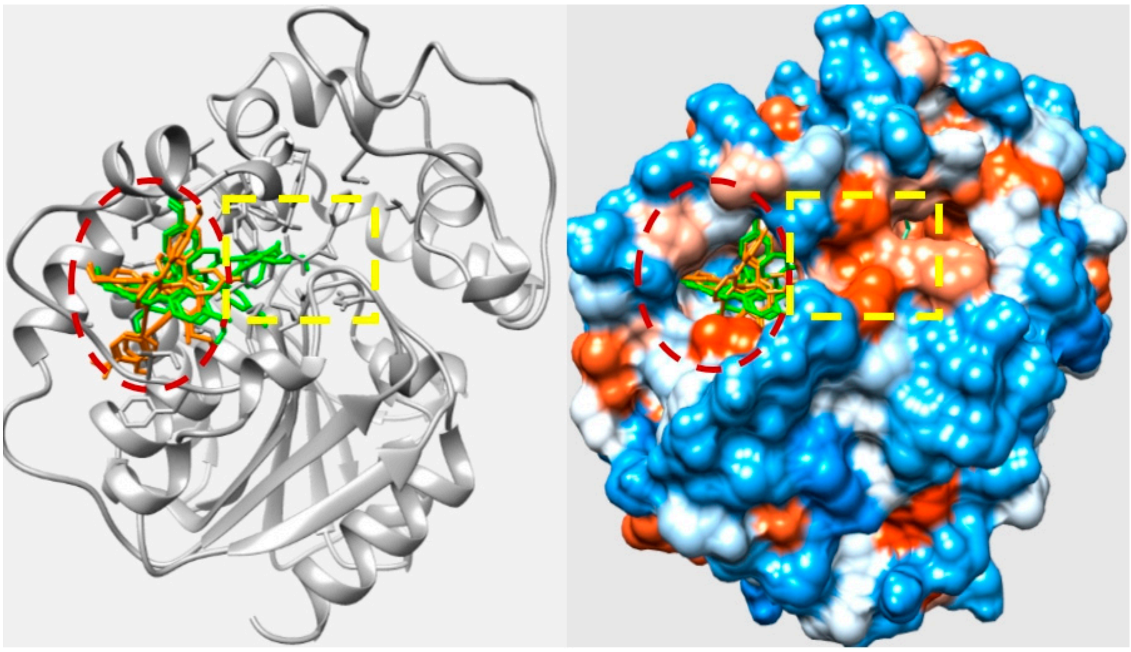

Figure S12. The best binding pose of **1**–**3** (green) with free enzyme and **1**–**3** (orange) with enzyme-substrate. Yellow box is the activity sites. Red ring is predicted binding site (pocket A).

Table S1. Validation data of selaginellins (**1**–**3**); linearity (R^2), calibration curve, limit of detection (LOD) and limit of quantitation (LOQ).

Compounds	Range (μg/mL)	Linearity (R^2)	Calibration Curve	LOD (μg/mL)	LOQ (μg/mL)
Selaginellin	0.6–100.0	0.9988	Y = 53.60x + 77.12	0.1	0.3
Selaginellin A	0.6–100.0	0.9992	Y = 21.55x + 26.81	0.1	0.3
Selaginellin B	0.6–100.0	0.9989	Y = 12.52x + 16.06	0.2	0.6

Table S2. Validation data of selaginellins (**1**–**3**); precision (intra-/inter-day) and accuracy (intra-/inter-day).

Compounds	Conc. (μg/mL)	Intra-Day (%, n = 5)		Inter-Day (%, n = 5)	
		Precision	Accuracy	Precision	Accuracy
Selaginellin	2.0	0.5	100.5	0.7	100.2
	6.0	0.4	101.2	0.5	101.3
	25.0	0.4	102.5	0.5	105.4
Selaginellin A	2.0	2.1	96.8	0.9	99.0
	6.0	0.3	96.2	0.7	96.1
	25.0	0.4	98.3	0.5	102.0
Selaginellin B	2.0	1.8	96.9	1.1	99.8
	6.0	0.6	98.5	1.6	97.6
	25.0	0.3	98.2	1.0	100.7
